# Simultaneous Detection of Group A Rotavirus in Swine and Rat on a Pig Farm in Brazil

**DOI:** 10.1155/2013/648406

**Published:** 2013-05-16

**Authors:** Paloma de Oliveira Tonietti, Aline Santana da Hora, Fernanda Dornellas F. Silva, Karen Linares Ferrari, Paulo Eduardo Brandão, Leonardo José Richtzenhain, Fabio Gregori

**Affiliations:** Department of Preventive Veterinary Medicine and Animal Health, College of Veterinary Medicine, University of São Paulo, Avenida Professor Dr. Orlando Marques de Paiva 87, 05508-270 São Paulo, SP, Brazil

## Abstract

This study investigated the occurrence of rotavirus in porcine and *Rattus norvegicus*, at the same time, on a pig farm in the city of Jaguariúna, São Paulo, Brazil. Swine (*n* = 21) and rat (*n* = 6) fecal samples were analyzed by nested RT-PCR assay. Rotavirus occurred in seven porcine and two rat samples. A total of three pig and one rat samples were further submitted to genetic sequencing. The partial NSP5 gene phylogeny showed that all strains were segregated in the genotype H1. These results point toward a cross-species transmission between rats and pigs on the surveyed farm and represent the first detection of rotavirus in *Rattus norvegicus* in Brazil.

## 1. Introduction

Rotaviruses are one of the most frequently detected viral agents associated with diarrhea in different animal species [1, 2]. Porcine rotavirus has been described in several countries such as Ireland [3], Germany [4], Slovenia [5], Russia [6], and Brazil [7], mostly affecting animals during neonatal and preweaning stages [2, 6]. Data about rotavirus occurrence in *Rattus norvegicus* is not common. A virus morphologically identical to typical rotaviruses, but antigenically distinct, was previously reported in suckling rats associated with infectious diarrhea of infant rats (IDIRs) [8]. Later, an identification of these viral structural proteins proved to be group B rotavirus [9]. In addition to that, rats have been used as animal model to study pathophysiology of rotavirus infection [10–13]. 

*Rotavirus* belongs to Reoviridae family and has a genome composed of 11 segments of double-stranded RNA [1, 14] that encodes six structural viral proteins (VP) (VP1, VP2, VP3, VP4, VP6, and VP7) and six nonstructural (NS) proteins (NSP1–NSP6). NSP5 is a phosphorylated and O-glycosylated serine- and threonine-rich protein of 198 amino acids involved in kinase activation [15], and it is an essential viroplasm component [14]. This protein is also crucial to the rotavirus replication cycle [16], and there are currently eleven (H1–H11) NSP5 genotypes [17]. The aim of the present study was to demonstrate the spatial, temporal, and genetic associations of rotavirus occurrence in pigs and rats on a commercial farm.

## 2. Materials and Methods

The study protocol was approved by the Institutional Animal Care and Use Committee of University of São Paulo.

The bovine group A rotavirus strain Nebraska calf diarrhea virus (NCDV) grown in MA-104 (green monkey fetal kidney) cells was used as a positive control, and 0.1% diethyl-pyrocarbonate-(DEPC-) water was treated as negative control in every four samples tested for all RNA-based procedures in order to monitor carryover contamination. Twenty-one fecal samples from pigs in neonatal and preweaning stages and six samples from the intestinal contents of *Rattus norvegicus* were collected at the same time from a pig farm in the city of Jaguariúna, São Paulo State, Brazil. Samples were prepared as 50% (p/v) suspensions in DEPC-treated water, clarified at 12000 ×g for 15 min at 4°C. Total RNA extraction from reference virus and the supernatants of the field samples were carried out with TRIzol reagent (Invitrogen, Carlsbad, CA, USA) and cDNA was synthesized using Random Primers (Invitrogen, Carlsbad, CA, USA) and M-MLV Reverse Transcriptase (Invitrogen, Carlsbad, USA) as indicated by manufacturer. 

Rotavirus screening was carried out with the nested RT-PCR [6], using Platinum Taq DNA Polymerase (Invitrogen, Carlsbad, CA, USA) according to the manufacturer's instructions. Positive samples presenting 317 bp under conventional gel electrophoresis were purified with ExoSAP-IT PCR Product Cleanup (USB Products Affymetrix, Cleveland, CA, USA) and submitted to bidirectional DNA sequencing with BigDye 3.1 (Applied Biosystems, Carlsbad, CA, USA), according to the manufacturer's protocols. Each step was carried out in separate rooms with separate materials. Nucleotide sequences were defined using a 3500 Genetic Analyser (Applied Biosystems, Carlsbad, CA, USA). The NSP5 gene sequences were aligned with homologous sequences from different rotavirus genotypes retrieved from GenBank with CLUSTAL/W 2.1 [18], and a phylogenetic tree was generated with the neighbor-joining distance algorithm and maximum composite likelihood substitution model with 1,000 bootstrap replicates using Mega 5.05 software [19]. 

## 3. Results

A total of seven porcine and two rat fecal samples were found positive by nested RT-PCR reaction. No amplifications were detected among the negative controls. Sequence analysis of three swine and one rat PCR amplicons (GenBank accession numbers JX626241–JX626244) obtained in this study confirmed the PCR findings and revealed a nucleotide identity ranging from 97.8% to 100% and the amino acid identity between 98.8% and 100% within a 259 bp stretch. A single amino acid mutation on aa 37 (using as reference strain PRG921, accession number JF796711) presented as serine and asparagine, respectively, in pig and rat samples.

## 4. Discussion

**Figure 1:**
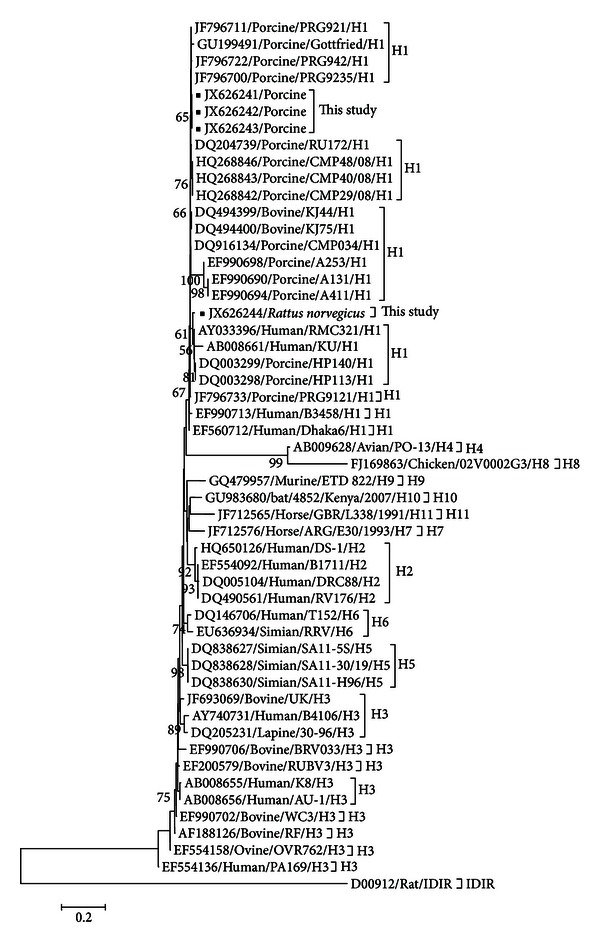
Nucleotide neighbor-joining distance tree (maximum composite likelihood model) for the partial NSP5 rotavirus gene showing the known genotypes for this gene. Pig and rat strains in the present study are preceded by black squares. The numbers at each node are bootstrap values (1,000 replicates). The bar represents the number of substitutions per site.

Although group B rotavirus has already been described in rats [9] and pigs in Brazil [20], the submission of the NSP5 gene fragments generated in this study to BLAST/n at http://www.ncbi.nlm.nih.gov/BLAST confirmed group A rotavirus identity relatedness. A stretch of 259 nucleotides of NSP5 encoding gene residues, related to the N-terminal portion of the protein [16], was used to build a phylogenetic tree, on which the genotype segregation (H1–H11) was maintained [17], as shown in Figure 1. 

Nucleotide sequences comparison revealed that the porcine and rat strains have a high degree of nucleotide sequence identity. The swine strains demonstrated the highest identity (99.6%) with porcine strains PRG9235 and PRG921 (JF796700 and JF796711, resp.). The rat strain showed the highest similarity (98%) with the porcine strains HP140 and HP113 (DQ003299 and DQ003298, resp.) but only 47.6% with the rat rotavirus IDIR (accession number D00912). These data suggests cross-species transmission of group A rotavirus between rats and pigs on the surveyed farm. 

Evidence to support the hypothesis that there is a dynamic interaction between rotavirus of human and animal origins, particularly in pig and cattle, was reported [2]. Moreover, all Brazilian pig and rat samples were clustered into H1 genotype, frequently described elsewhere in pigs and humans [21–23]. In conclusion, to our knowledge, this is the first detection of rotavirus in *Rattus norvegicus* in Brazil, and this animal may pose as a risk for transmission and maintenance of the virus circulation on the pig farms as well as other animal species.
